# The Glycine-Rich Domain Protein GRDP2 Regulates Ovule Development via the Auxin Pathway in *Arabidopsis*

**DOI:** 10.3389/fpls.2021.698487

**Published:** 2021-10-29

**Authors:** Lulu Wang, Yanhui Liu, Mohammad Aslam, Bello Hassan Jakada, Yuan Qin, Hanyang Cai

**Affiliations:** ^1^Guangxi Key Laboratory of Sugarcane Biology, State Key Laboratory for Conservation and Utilization of Subtropical Agro-Bioresources, College of Agriculture, Guangxi University, Nanning, China; ^2^College of Life Sciences, Fujian Agriculture and Forestry University, Fuzhou, China

**Keywords:** *GRDP2*, female gametophyte, ovule development, auxin, embryo sac

## Abstract

The glycine-rich domain proteins (*GRDP*) have been functionally implicated in the cell wall structure, biotic, and abiotic stress responses. However, little is known about *GRDP* genes in female gametophyte development of *Arabidopsis*. This study shows that *GRDP2*, a *GRDP*, plays a crucial role in female gametophyte development. In *GRDP2* overexpression lines, *grdp2-3*, the embryo sacs were arrested at FG1 and no nucleus stages. Furthermore, callose staining shows that cell plate formation during megasporogenesis is disturbed in *grdp2-3*. In contrast, the pollen development is not affected in *grdp2-3*. The expression patterns of auxin-specific marker lines in female gametophytes showed that the auxin distribution and transport were significantly changed during megagametogenesis in *grdp2-3*. In addition, compared with the membrane-localized pattern of *PIN1*, *PIN2*, and *PIN7* in WT, the signals were detected in the cytoplasm in *grdp2-3*. Together, our data suggest that *GRDP2* plays an essential role in auxin-mediated female gametophyte development.

## Introduction

Proteins with particular glycine-rich regions have been reported in seed coat and cell wall of a wide variety of plants; for example, soybean seed coat contains 21% of glycine, the cell wall of milkweed stem has 31%, and coat coleoptile cells have 27% of glycine (de Oliveira et al., 1990). Glycine-rich proteins (GRPs) have also been isolated from pumpkin seed coat (Varner and Cassab, 1986) and strawberry fruit (Reddy and Poovaiah, 1987) with approximately 47% and 49% glycine residues, respectively. GRP proteins contain characteristic repetitive glycine stretches, amino-terminal sequences characterized by a high content of glycine (∼40–70%), and a repetitive sequence of residues arranged in (Gly)n-X motifs (Sachetto-Martins et al., 2000; Mousavi and Hotta, 2005). At present, five groups of GRPs [i.e., (I) (GGX)_*n*_, (II) (GGXXXGG)_*n*_, (III) (GXGX)_*n*_, (IV) glycine-rich domain with additional motifs such as RNA-recognition motif (RRM) or a cold-shock domain (CSD), CCHC zinc-fingers and (V) GGX/GXGX] have been suggested based on the general structure and the arrangement of the repeated glycine signatures as well as the presence of conserved motifs and domains (Bocca et al., 2005; Mangeon et al., 2010; Ortega-Amaro et al., 2014).

Glycine-rich proteins genes exhibit tissue-specific expression patterns and regulate many developmental processes in plants. Previous studies suggest that GRPs are specifically expressed in the xylem, phloem, epidermis, anther tapetum, and roots (Fusaro and Sachetto-Martins, 2007). GRPs are generally induced by physical, chemical, and biological factors. GRPs function in the plant cell wall structure, plant defense response, regulation of plant flowering time, transcriptional regulation, signal transduction, protein–protein interaction, stress response, etc. (Keller et al., 1988; Bocca et al., 2005; Mangeon et al., 2010; Ortega-Amaro et al., 2014). The PvGRP18, a glycine-enriched protein in legumes, was reported as an agglutinant for cell wall synthesis which is involved in protoxylem growth (Keller et al., 1988; Ryser et al., 1997). PtGRP1 is located between the cell wall and cell membrane, and its expression level in stem and leaf decreases with the increase of tissue development age (Condit, 1993). Phytohormones control several aspects of plant growth, development, and stress response, also regulate GRP expression. For example, GRPs accumulate significantly in auxin deficient strawberry fruits (Reddy and Poovaiah, 1987). In pea, GRP1 gets enriched in fruits and seeds; however, ABA induction results in its increase in the pistil and root (Urbez et al., 2006). Overexpression of alfalfa GRP leads to salt and ABA sensitivity in *Arabidopsis thaliana* (Long et al., 2013).

In Eucalyptus, apart from the canonical GRPs, another type, the glycine-rich domain (GRDPs), was reported (Bocca et al., 2005; Rodríguez-Hernández et al., 2014). GRDPs have a short glycine-rich domain located in the C terminal of the protein along with repeated GCGXXCXGXCG. Besides, a comparison of GRDPs in 16 species indicated two additional conserved domains in this protein: Domain of Unknown Function (DUF1399) at the N-terminal and a putative RNA binding motif (RNP) in the middle of the protein sequence. In *Arabidopsis*, there are two members of the GRDPs, *GRDP1* and *GRDP2*. The glycine-rich domain (GRD) is located at the C-terminus of the GRDP1 and GRDP2 proteins, with five glycine regions in the consensus regions XSGCGXXCXGXCG (GRDP1) or GCGXXCXGXCGXXCG (GRDP2) (Rodríguez-Hernández et al., 2014). Besides, two highly conserved domains, DUF1399 at the N-terminus and the putative RNP in the middle, which were also detected in GRDPs. Previously, it was reported that *GRDP1* plays a regulatory role in ABA signaling and abiotic stress tolerance (Rodríguez-Hernández et al., 2014), whereas *GRDP2* regulates development and stress responses possibly through an auxin-dependent mechanism in *Arabidopsis*, and common bean (Ortega-Amaro et al., 2014, 2016; Rodríguez-Hernández et al., 2014).

Phytohormones such as auxin, gibberellin, cytokinin, ABA, ethylene, and brassinosteroids play vital roles in plant reproduction. They regulate the development of both male and female reproductive organs, including ovules and gynoecia (Nambara and Van Wees, 2021). For example, auxin is involved in regulating the development of male and female reproductive organs of plants, including ovule and pistil (Liu et al., 2020). It plays an essential role in developing seeds and fruits (Dorcey et al., 2009; Figueiredo et al., 2016). Auxin response is mediated by a series of auxin response factors (ARFs) that bind to specific auxin response elements (AUXRE) in the promoters of their target genes (Ulmasov et al., 1997; Roosjen et al., 2018). At low auxin concentrations, responses are blocked by Aux/IAA repressor proteins, which form dimers with activating ARFs, thus preventing them from regulating their target genes. When auxin levels rise, the phytohormone facilitates binding between the DII domain of the Aux/IAA proteins and TIR1/AFB F-box proteins that are part of an E3 ubiquitin ligase complex (Larsson et al., 2017), resulting in the targeting of the Aux/IAAs for degradation by the 26S proteasome, thereby releasing the ARFs from repression (Roosjen et al., 2018). In 2004, a group of genes regulated explicitly by indole-3-acetic acid (IAA) had been identified through gene microarray, including *GRDP2* (Goda et al., 2004). Therefore, it is speculated that the auxin signaling could regulate the expression of the GRDP2 gene.

In this study, we performed molecular characterization of *GRDP2* (*At4g37900*), both *p35S:GRDP2* and *pUBQ10:GRDP2* overexpressing lines and *grdp2*-3 mutants were analyzed. Expression profiles analysis of the *GRDP2* gene in *Arabidopsis* showed that it is highly expressed in floral organs. We further showed that *GRDP2* overexpression results in two types of ovule development defects in *Arabidopsis thaliana*, the abnormal arrest at one nucleus stage (FG1 stage) and the nucleus degeneration. Auxin distribution analysis during the gametogenesis in *grdp2-3* ovules suggests that auxin may play an essential role in *grdp2-3* ovule development.

## Materials and Methods

### Plant Material and Growth Conditions

The mutant and transgenic lines used in this study were generated in the *Arabidopsis thaliana* ecotype Columbia 0 (Col-0) background. *Arabidopsis* seeds of each line were surface-sterilized for 10 min with 75% ethanol, 5 min with absolute ethyl alcohol, and rinsed five times in sterile distilled water. Aseptic stratified seeds (2 days at 4°C) were germinated and grown on agar plates containing 1/2 Murashige and Skoog (MS) medium, pH = 5.7, 0.5% (w/v) sucrose, and 0.8% (w/v) agar (Murashige and Skoog, 1962). Plates were incubated in a growth chamber with a photoperiod of 16 h/8 h, light/dark cycle at a temperature of 22 ± 1°C. Afterward, plants were transferred to soil pots in a growth chamber at 22 ± 1°C with a 16 h light/8 h dark photoperiod (Zhao et al., 2014). For the root growth transfer experiment, WT and mutants’ seeds were sterilized and planted on Hoagland media as detailed previously (Aslam et al., 2020). After 5 days, plants were transferred to Hoagland media supplemented with different IAA or NPA concentrations (15 nM and 30 nM). The plates were then marked, and after 48 h of vertical culture, root length was recorded.

### Identification of the T-DNA Insertion Lines and RT-qPCR

Three T-DNA mutant lines Sail_387_D04, SALK_412A10, and SALK_112794C for the *GRDP2* (At4g37900) gene were acquired from the Arabidopsis Biological Resource Center^1^. *pDR5:GFP*, *pPIN1:PIN1-GFP*, *pPIN2:PIN2-GFP*, *pPIN7:PIN7-GFP*, and *pYUCCA5-GFP-GUS* (from Dr. Xu Chen, Fujian Agriculture and Forestry University, Fuzhou, China). *pSPL:GUS*, *pKNU:KNU-VENUS*, *pAKV:H2B-YFP*, *pDD45:GFP*, and *pDD65:GFP* (from Dr. Yuan Qin, Fujian Agriculture and Forestry University, Fuzhou, China). The T-DNA insertion was identified by primers (Supplementary Table 1) obtained from the SIGnAL website^2^. *GRDP2* expression level in T-DNA mutant lines was confirmed by RT-qPCR using the primers listed in Supplementary Table 1.

Total RNA was extracted from inflorescence tissues using the RNA extraction Kit (Omega Bio-Tek, Shanghai, China) following the manufacturer’s protocol. EasyScript^®^ cDNA Synthesis SuperMix (Transgen, Beijing, China) was used for the cDNA preparation. The RT-qPCR was conducted using TransStart^®^ Top Green qPCR SuperMix (Transgen, Beijing, China). *HK2* (AT5G35750) gene was used as a reference gene (Supplementary Table 1; Zhao et al., 2018). These assays were conducted for three biological replicates, and the results are shown as the mean ± standard deviations.

### Histological Analysis of Female Gametophyte Development

Differential interference contrast (DIC) microscopy was used for the histological analysis of *grdp2* mutant ovule development. The whole inflorescences of *grdp2* and WT plants were fixed with FAA solution (6 alcohol:3 chloroform:1 acetic acid) for more than 16 h at room temperature. Then siliques were then dissected longitudinally with hypodermic needles (1 ml insulin syringes), and all the ovules were exposed outside the carpels. Finally, cleared ovules were observed on a Zeiss Axioimager M1 microscope (Carl Zeiss Canada, Toronto, ON, Canada) under DIC optics, and images were acquired with a Zeiss AxioCam MR digital camera. All images were processed for publication using Adobe Photoshop (Adobe Systems, San Jose, CA, United States).

### Vector Construction and Plant Transformation

Using a high-fidelity polymerase kit (QIAGEN, United States), the open reading frame of *GRDP1* and *GRDP2* was amplified from an inflorescence cDNA sample using gene-specific primers, and the *AtGRDP1* and *AtGRDP2* promoter region (2 kb upstream sequence) was amplified from the *Arabidopsis* genomic DNA (Supplementary Table 1). The PCR fragments were verified by DNA sequencing and then cloned into the pENTR/D-TOPO vector (Invitrogen, Dusseldorf, Germany). Then the promoter fragments were recombined into the destination vector pGWB633, and pGWB604; and the CDS were recombined into the destination vector pGWB602 (*p35S:GRDP2-OE*), and pGWB501 (*pUBQ10:GRDP1-OE*; *pUBQ10:GRDP2-OE*), using LR Clonase II (Invitrogen, Thermo Fisher Scientific, CA, United States). The plasmids were extracted using the E.Z.N.A.^®^ Plasmid Maxi Kit (Omega Bio-Tek, Norcross, GA, United States) following the manufacturer’s procedure.

An *Agrobacterium tumefaciens* strain (GV3101) carrying the fragments as described before was introduced into *Arabidopsis thaliana* WT plants by the floral dip method (Clough and Bent, 1998). Transgenic plants were selected in soil by spraying with Basta (Basta:H_2_O = 1:1000) or on the MS media and selected by hygromycin. Multiple independent T2 transgenic lines were used for phenotype observation.

### Confocal Laser Scanning Microscopy

Dissected carpels were mounted with 40% glycerol. Confocal laser scanning microscopy (CLSM) analysis was performed using a Leica TCS SP8X Meta confocal microscope. To detect the GFP signal, a 488 nm wavelength laser was used for excitation, and a BP 505–550 nm filter was applied for GFP emission. For FM 4-64 FX dye application (Invitrogen), an additional BP 575–615 nm filter was used. For ovule morphological analyses, pistils were fixed as described earlier (Braselton et al., 1996). Samples were excited using a 532 nm laser, and emission was detected between 570 and 740 nm.

### Alexander Staining of Pollen Grains

Mature flower buds were collected before anthesis, and non-dehiscent anthers (stage 12 flower buds) were fixed in FAA (6 alcohol:3 chloroform:1 acetic acid) solution for a minimum of 2 h (Peterson et al., 2010). Then the buds were placed on a microscope slide and the fixative was removed with absorbent paper. Two to four drops of the stain solution were applied before the sample completely dries, and the buds were dissected to release the anthers. The sample in the stain was slowly heated on an alcohol burner in a fume hood until the stain solution reached near-boiling. The samples were washed three times with 40% glycerol and mounted for analysis using the ZEISS microscope (ZEISS, Germany).

### Selection of CRISPR/Cas9 Target Sites and Molecular Analysis and Detection of Mutations

All possible CRISPR target sites within *GRDP1* and *GRDP2* are identified with the online tool SSC^3^. Then three candidates of CRISPR target sites were selected based on the genomic location and potential off-target scores, and the sgRNA targets two target genes simultaneously. Each of the targeting portions was synthesized as a pair of reverse complementary oligonucleotides, then assembled the natural fading primers into the pAtU6-M vector by T4 ligase (Thermo Fisher, Waltham, MA, United States). assembled into the pCambia1300-UBQ: Cas9-P2A-GFP-rbcS-E9t vector by GoldenGate reaction assembly (NEB, Hitchin, United Kingdom). These constructs were confirmed by DNA sequencing and transformed into *Arabidopsis* by *Agrobacterium* strain GV3101 separately.

The primers of hygromycin were used to identify transgenic plants containing the T-DNA fragment fused into the genome (Supplementary Table 1). The positive plants were deep sequenced to identify any insertions or deletions within the targeted region. The phenotypes of the transgenic plants were photographed and described in section “Results.”

## Results

### Overexpression of *GRDP2* Causes Reduced Plant Fertility

**FIGURE 1:**
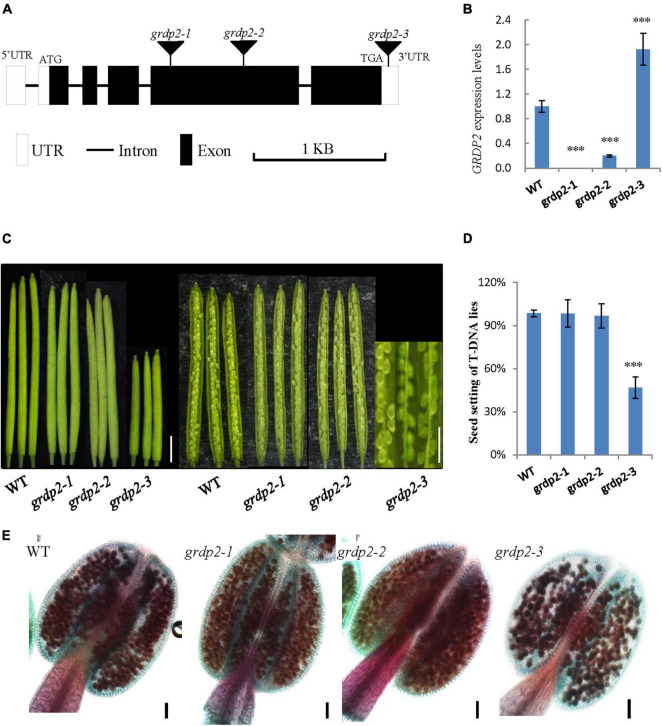
Phenotype analysis of *grdp2* T-DNA mutant. **(A)** Gene structure of *GRDP2* and T-DNA insertion sites of the mutant alleles in *grdp2*. White boxes show UTRs; gray lines show introns; black boxes show exons. **(B)** Expression level of the three T-DNA insertion lines as determined by real-time RT-PCR. **(C)** Seed development in wild-type (WT) plants and the *grdp2* mutant alleles. Bar = 1 cm. **(D)** Seed setting statistics of three T-DNA insertion lines. **(E)** Alexander’s staining of pollen from WT and three *grdp2* mutant alleles. The red color indicates viable pollen. Bar = 50 μm. The asterisks show the significance level (^∗∗∗^*p* < 0.01) as judged by the Student’s *t*-test.

In *Arabidopsis*, glycine-rich domain protein (*GRDP*) is encoded by a single copy gene *GRDP2*, and its genomic organization consists of five exons and four introns. To investigate the function of *GRDP2* in *Arabidopsis*, we obtained three independent T-DNA inserted alleles of *GRDP2* from the Arabidopsis Biological Resource Center (ABRC); *grdp2-1* (SALK_387D04), *grdp2-2* (SALK_412A10), and *grdp2-3* (SALK_112794C). The T-DNA insertion site of these mutants is shown in Figure 1A. The *GRDP2* expression level of these mutants was quantified through the RT-qPCR. The results showed that *grdp2-1* is a knockout line and *grdp2-2* is a knockdown line, while the *GRDP2* expression is much higher than WT plants in *grdp2-3* (Figure 1B). Phenotypic analysis of the *grdp2* T-DNA mutant lines showed a distinct low seed set (51%) of the *grdp2*-3 line with short siliques than WT (Figures 1C,D).

We conducted a genetic analysis to determine whether the mutation of *GRDP2* caused gametophyte sterility or embryo lethality. A reciprocal cross experiment showed that *grdp2-3* has 54.48% sterile or aborted ovules when *grdp2-3* was used as the mother plant and pollinated by WT pollen. Conversely, when WT pistils were pollinated with *grdp2-3* pollen, only 8.84% of ovules were abnormal (Table 1). This result indicated that the female gametophyte development has a problem, and the pollen development is normal, as shown by Alexander’s staining of pollen grains (Figure 1E). These results indicate that the abnormal development of female gametophytes causes the reduced seed setting rate of *grdp2-3*.

**TABLE 1:** Analysis of seed development in siliques from selfing and reciprocal crosses of *grdp2-3* plants.

**Female × Male**	**Total seeds**	**Abnormal seeds**	**Abortion rate**
+/+ × +/+	521	16	2.98%
*grdp2-3^– – –^* × *grdp2-3^–^*^/^*^–^*	432	214	49.54%
*grdp2-3^–^*^/^*^–^* × +/+	714	389	54.48%
+/+ × *grdp2-3^–^*^/^*^–^*	790	70	8.86%

*Reciprocal crosses between wild-type and *grdp2-3* homozygous plants. The crosses exhibited reduced fertility only when *grdp2-3* plants were used as female parents, indicating the reduced fertility phenotype is determined by the lack of *GRDP2* function in the female gametophyte. +/+, wide type.*

### Differential Expression Patterns of *GRDP2* in *Arabidopsis*

**FIGURE 2:**
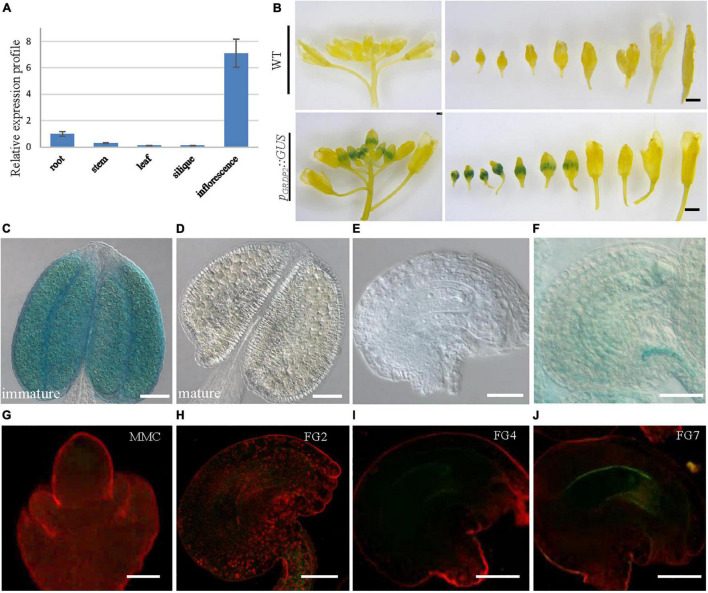
The expression profiles of *GRDP2* in *Arabidopsis.* **(A)** Real-time PCR to quantify *GRDP2* expression levels in different tissue of root, stem, leaf, siliques, and inflorescence. **(B)** *pGRDP2:GUS* expression pattern in floral buds. Bars = 1 mm. **(C,D)** *pGRDP2:GUS* expression pattern in anthers. Bars = 50 μm. **(E,F)** *pGRDP2:GUS* expression pattern in ovules. Bars = 20 μm. **(G–J)** *pGRDP2:GFP* expression pattern in different ovule development stages. The developmental stages of the ovules are indicated at the top right corner of each panel. Bars = 20 μm.

To better study the functions of *GRDP2*, its expression pattern was observed in root, stem, leaf, siliques, and inflorescence tissues using quantitative PCR. The result showed that *GRDP2* has a higher expression in inflorescence than root, stem, leaves, and siliques (Figure 2A). We then fused the *GRDP2* promoter with GFP/GUS protein to detect the expression patterns of *GRDP2* in inflorescence tissue. GUS expression was detected in the whole inflorescence. However, only immature flowers showed the GUS signals, especially in the young pollen grains (Figures 2B–D). GUS signal was also detected in ovules during the female gametophyte development, and no GUS signal was detected at early stages (Figure 2E). A weak GUS signal was detected in the embryo sac and funiculus of ovules at the mature stage (Figure 2F).

Similarly, the GFP reporter gene was also used to visualize the expression pattern of *GRDP2*. No GFP signals were detected in the ovules during the megasporogenesis stage and early megagametogenesis stage (Figures 2G–I). The mature FG7 ovules expressed a weak GFP signal (Figure 2J), suggesting *GRDP2* express only in mature ovules.

### Overexpression of *GRDP2* Results in Abnormal Ovule Development in *Arabidopsis thaliana*

**FIGURE 3:**
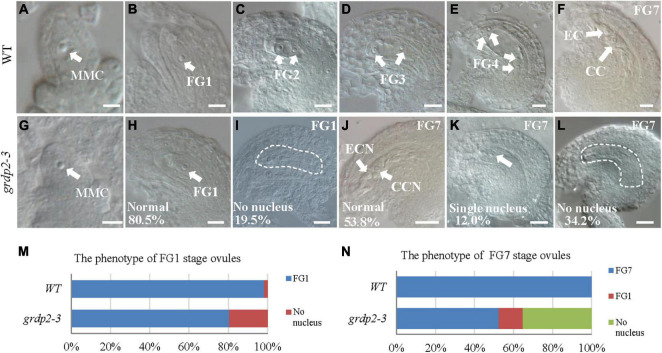
Female gametogenesis in WT and *grdp2-3* plants examined by differential interference contrast microscope (DIC). Bar = 50 μm. **(A)** Megaspore mother cell (MMC) of WT plants. **(B)** 98.3% of 121 examined FG1 stage from WT normal ovules. **(C–F)** Female gametogenesis from FG2 to FG7 was normal in WT plants. **(G)** Megaspore mother cell (MMC) of *grdp2-3* plants. **(H)** 80.5% of 221 examined FG1 stage ovules from *grdp2-3* plants were normal. **(I)** 19.5% of 221 examined FG1 stage ovules from *grdp2-3* plants showed no nucleus. **(J)** 53.8% of 179 examined FG7 stage ovules from *grdp2-3* plants were normal. **(K)** 12.0% 179 examined FG7 stage ovules from *grdp2-3* plants showed single nucleus. **(L)** 34.2% of 179 examined FG7 stage ovules from *grdp2-3* plants showed no nucleus. **(M)** The phenotype analysis of FG1 stage ovules in WT and *grdp2-3* plants. **(N)** The phenotype analysis of FG7 stage ovules in WT and *grdp2-3* plants. CCN, central cell nuclei; EC, egg cell nuclei; FG, female gametophyte; white arrows mean nuclei; white dotted lines mean embryo sac.

To further uncover the underlying mechanisms of female gametophyte development defects, we investigated the female gametophyte structure in WT and *grdp2-3* mutants via DIC microscopy. In general, female gametophyte development is divided into two main steps, megasporogenesis and megagametogenesis (Schneitz et al., 1995; Christensen et al., 1997). During megasporogenesis, the megaspore mother cell (MMC) (Figure 3A) undergoes meiosis and produces four haploid megaspores. Three of the megaspores degenerates, and only one persists to become the functional megaspore (FM) (Figure 3B). Subsequently, the FM undergoes three consecutive mitotic divisions (FG1–FG4), nuclear fusion, and cellularization (FG5–FG6) that lead to the formation of the mature embryo sac (FG7) (Figures 3C–F). In the FG7, WT female gametophyte consists of a central cell and an egg cell (Figure 3F).

In contrast, 34.2% of mutant ovules exhibited an absence of the female gametophyte, and 12.0% of mutant ovules have only one abnormal nucleus (Figures 3J–L,N) that may consequently cause an inability of the pollen tube to penetrate the ovule leading to abortion. We examined the female gametophyte structure of the *grdp2-3* mutant plants at the early development stages to identify the exact stage of development defects. The result showed normal MMC (Figure 3G), while 19.5% of the functional megaspore produced after meiosis were abnormal and resulted in non-nucleus ovules (Figures 3H,I,M). These results suggest that defects in the mutant manifest during female meiosis before the functional megaspore is formed.

**FIGURE 4:**
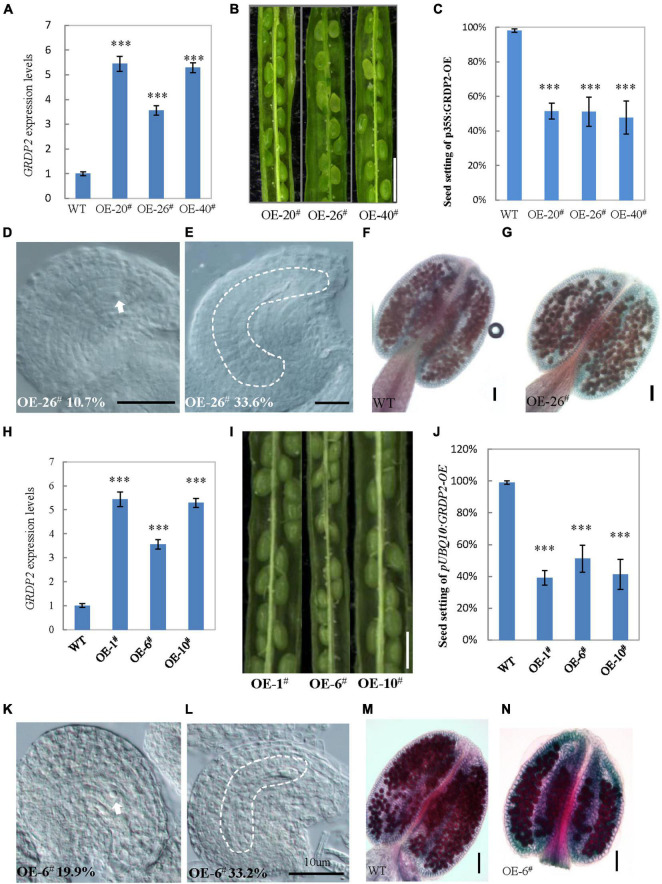
Phenotype analysis of *p35S:GRDP2*-OE and *pUBQ10:GRDP2*-OE lines. **(A)** Expression levels of *GRDP2* in WT, *p35S:GRDP2-OE-20^#^*, *p35S:GRDP2-OE-26^#^*, and *p35S:GRDP2-OE-40^#^* as determined by real-time RT-PCR. **(B)** Seed development in three *p35S:GRDP2-OE* line plants. Bar = 1 cm. **(C)** Seed setting statistic of the WT, *p35S:GRDP2-OE-20^#^*, *p35S:GRDP2-OE-26^#^*, and *p35S:GRDP2-OE-40^#^* lines. **(D,E)** 10.7% of 316 examined FG7 stage ovules from *p35S:GRDP2-OE-26^#^* line plants showed single nucleus **(D)**, 33.6% of 316 examined FG7 stage ovules from *p35S:GRDP2-OE-26^#^* line plants showed no nucleus **(E)**. **(F,G)** Alexander’s staining of pollen from WT **(F)** and *p35S:GRDP2-OE-26^#^* **(G)** line plants. The red color indicates viable pollen. Bar = 50 μm. **(H)** Seed development in wild-type (WT) and three *pUBQ10:GRDP2-OE* line plants. Bar = 1 cm. **(I)** Expression levels of GRDP2 in WT, *pUBQ10:GRDP2-OE-1^#^*, *pUBQ10:GRDP2-OE-6^#^*, and *pUBQ10:GRDP2-OE-10^#^* as determined by real-time RT-PCR. **(J)** Seed setting statistics of the WT, *pUBQ10:GRDP2-OE-1^#^*, *pUBQ10:GRDP2-OE-6^#^*, and *pUBQ10:GRDP2-OE-10^#^* lines. **(K,L)** The FG7 stage ovules from *pUBQ10:GRDP2-OE-26^#^* line plants. **(M,N)** Alexander’s staining of pollen from WT **(M)** and *pUBQ10:GRDP2-OE-6^#^* **(N)** line plants. The red color indicates viable pollen. Bar = 50 μm. Vertical bars represent the mean ± SD as determined by two technical replicates of three biological replicates; asterisks show the significance level judged by Student’s *t*-test (^∗∗∗^*p* < 0.01). White dotted lines mean embryo sac.

The expression analysis and *GRDP2* phenotype in different T-DNA lines (Figure 1B) suggested that overexpression of *GRDP2* results in abnormal ovule development. Therefore, we overexpressed *GRDP2* and verified our results using *p35S:GRDP2-*OE and *pUBQ10:GRDP2*-OE overexpression lines. The positive homozygous plants of the T2 generation were analyzed for their phenotype. Expression of the *GRDP2* gene was significantly up-regulated in three randomly selected independent lines of overexpression plants (Figures 4A,H). The seed setting was decreased considerably (about 50%) in the GRDP2 overexpressing lines (Figures 4B,C,I,J). Further phenotypic observation of the ovules of *p35S:GRDP2-OE-26^#^*, and *pUBQ10:GRDP2-OE-6^#^* plants at the stage of FG7 showed that there were two types of abortions; 30% of the ovules had no embryo sac, and 15% of the ovules were arrested at FG1 stage (Figures 4D,E,K,L). Consistent with the phenotype of *grdp2-3* T-DNA insertion plants, Alexander’s staining of pollen showed that the pollen development in these overexpression plants was similar to WT (Figures 4F,G,M,N). Taken together, these results suggest that the accumulation of *GRDP2* in *Arabidopsis* results in abnormal ovule development.

### Megasporogenesis Is Affected in *grdp2-3* Mutants

**FIGURE 5:**
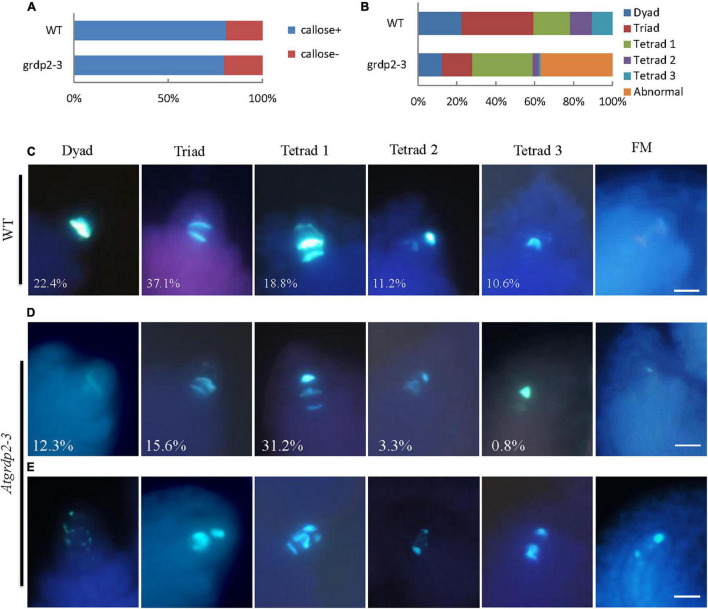
Callose deposition during megasporogenesis are defective in *grdp2-3* mutant ovules. **(A)** Quantification of callose staining-positive (+) and callose staining-negative (–) ovules at stage 2IV in more than 100 ovules for each sample. **(B)** Quantitative profile of callose deposition at various stages of meiosis in more than 100 ovules for each sample. **(C)** Callose-stained wall deposition in WT ovules at stage 2IV. **(D)** Callose-stained wall deposition in *grdp2-3* ovules at stage 2IV. **(E)** Abnormal callose staining is observed in *grdp2-3* ovules during meiosis.

As stated above that abnormalities were probably appearing in the meiosis of *grdp2-3* mutants. Therefore, we investigated whether female meiocytes in the *grdp2-3* ovules were competent to enter meiosis in the WT and *grdp2-3* ovules at stage 2-IV (Figure 5A). The result revealed that female meiocytes in the *grdp2-3* mutant entered meiosis similar to WT. Furthermore, to determine whether subsequent steps in meiosis were also affected in the *grdp2-3* mutant, we examined callose deposition during meiosis progression. The result showed that in WT ovule, the callose signal first appears at the cell plate that separates the two daughter cells of female meiocyte in a dyad (Figure 5C, Dyad), followed by accumulation at the cell plates that separate the cells of a triad and a tetrad (Figure 5C, tetrad 1). After meiosis, the callose signal disappeared from the cell plate separating the four megaspores forming tetrad 2 and tetrad 3 (Figure 5C, tetrad 2 and tetrad 3). When a functional megaspore was formed, the callose signal almost disappeared in WT (Figure 5C, FM). However, in the *grdp2-3* mutant, callose deposition during megasporogenesis differed from those observed in the WT ovules. *grdp2-3* exhibited abnormal callose staining (Figure 5E), suggesting that cell plate formation during megasporogenesis is distorted in the *grdp2-3* mutants. In addition to the aberrant callose-staining signal during the meiosis, we observed that callose fluorescence persisted in *grdp2-3* ovules, even at later development stages (Figure 5E, FM). An increase at triad 1 stage in the *grdp2-3* (31.2%) mutant compared to WT (18.8%) and a decrease in tetrad 2 (3.3%) and tetrad 3 (0.82%) (Figures 5B,D) were observed in the ovules at stage 2-IV, implying that the progression of meiosis is disturbed in the *grdp2-3* mutant ovules. Also, 36.9% of ovules exhibited abnormal callose staining, suggesting cell plate formation during megasporogenesis is affected in the *grdp2-3* mutants (Figures 5B,E).

### Female Gametophyte Specific Maker Genes Reveal Abnormal Ovule Development of *grdp2-3*

**FIGURE 6:**
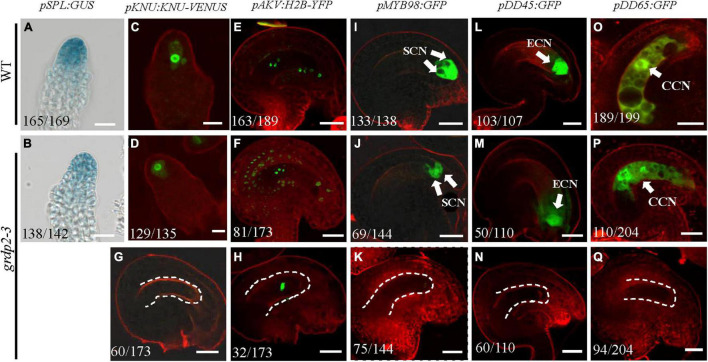
Expression of female gametophyte specific markers. **(A,B)** *pSPL:GUS* expression in **(A)** WT at stage 2-I ovules prior to entering meiosis (165/169) and **(B)** *grdp2-3* stage 2-I ovules (138/142). **(C,D)** *pKNU:KNU-VENUS* expression in **(C)** WT at MMC stage ovules (151/156) and **(D)** *grdp2-3* at MMC stage ovules (129/135). **(E)** *pAKV:H2B-YFP* expression in WT at FG6/FG7 stage ovules (163/189), the rest ovules are in the FG5 stage. **(F–H)** *pAKV:H2B-YFP* expression in **(F)** *grdp2-3* at FG6/FG7 stage ovules (81/173) ovules are same as the WT; **(G)** 60/173 ovules have no nucleus; and **(H)** 32/173 ovule have one nucleus. **(I)** *pMYB98:GFP* expression in WT at FG7 stage ovules (133/138). **(J,K)** *pMYB98:GFP* expression in *grdp2-3* at FG7 stage ovules **(J)** 69/144 ovules are same as the WT and **(K)** 75/144 ovules have no GFP signal. **(L)** *pDD45:GFP* expression in WT at FG7 stage ovules (103/107). **(M,N)** *pDD45:GFP* expression in *grdp2-3* at FG7 stage ovules **(M)** 50/110 ovules are same as the WT and **(N)** 60/110 ovules have no GFP signal. **(O)** *pDD65:GFP* expression in WT at FG7 stage ovules (189/199). **(P,Q)** *pDD65:GFP* expression in *grdp2-3* at FG7 stage ovules **(P)** 110/204 ovules are same as the WT and **(Q)** 96/204 ovules have no GFP signal. Bar = 25 μm. CCN, central cell nuclei; ECN, egg cell nuclei; SCN, synergid cell nuclei; white arrows mean nuclei; white dotted lines mean embryo sac.

Previous studies have reported various female gametophyte-specific marker genes to detect the developmental process in the female gametophyte, such as the *pSPL-GUS*, *pKNU:KNU-VENUS* (Payne et al., 2004), *pAKV:H2B-YFP* (Schmidt et al., 2011; Cai et al., 2021), *pMYB98-GFP* (Kasahara et al., 2005; Steffen et al., 2007), *pDD45-GFP* (Steffen et al., 2007), and *pDD65-GFP* (Steffen et al., 2007). We introduced the different marker lines in the *grdp2-3* background and then analyzed their expression in the wild-type and mutant backgrounds. *SPL* encodes a novel nuclear protein with limited homology to MADS-box transcription factors and has an early function during male and female sporogenesis. Therefore, we could see the GUS signal in the nucellus at the early gametogenesis stage in the WT background (Figure 6A); the signal in *grdp2-3* was the same as WT (Figure 6B). Compared to WT, the expression of MMC-specific marker *pKNU:KNU-VENU*S was also normal (Figures 6C,D) in *grdp2-3*. These results suggest that the female gametophyte development at an early stage is not affected in *grdp2-3*. *pAKV:H2B-YFP* is a nucleus marker gene during the mitosis process of female gametophyte development. In the WT background, center cell, egg cell, synergid cell, and antipodal cell were present in the embryo sac at the mature FG7 stage (86.4%, *n* = 189) (Figure 6E). However, in *grdp2-3* only 46.8% (*n* = 173) normal ovules were present (Figure 6F), and a large proportion of no-nucleus (34.7%, *n* = 173) (Figure 6G) and one nucleus ovules (18.5%, *n* = 173) (Figure 6H) were seen. Consistently, in WT background at FG7 stage, the synergid cell marker *pMYB98-GFP* (96.4%, *n* = 138), egg cell marker *pDD45-GFP* (96.3%, *n* = 107) and central cell marker *pDD65-GFP* (95.0%, *n* = 199) showed normal GFP signal in synergid cell, egg cell, and central cell, respectively (Figures 6I,L,O), while in *grdp2-3* mutant there were only 47.9% (*n* = 144), 45.5% (*n* = 110), and 53.9% (*n* = 204) ovules with GFP signal at FG7 stage (Figures 6J,K,M,N,P,Q). These results indicate that *grdp2* is involved in the female gametophyte development in *Arabidopsis*.

### Auxin Distribution During Gametogenesis Is Disrupted in *grdp2* Ovule

**FIGURE 7:**
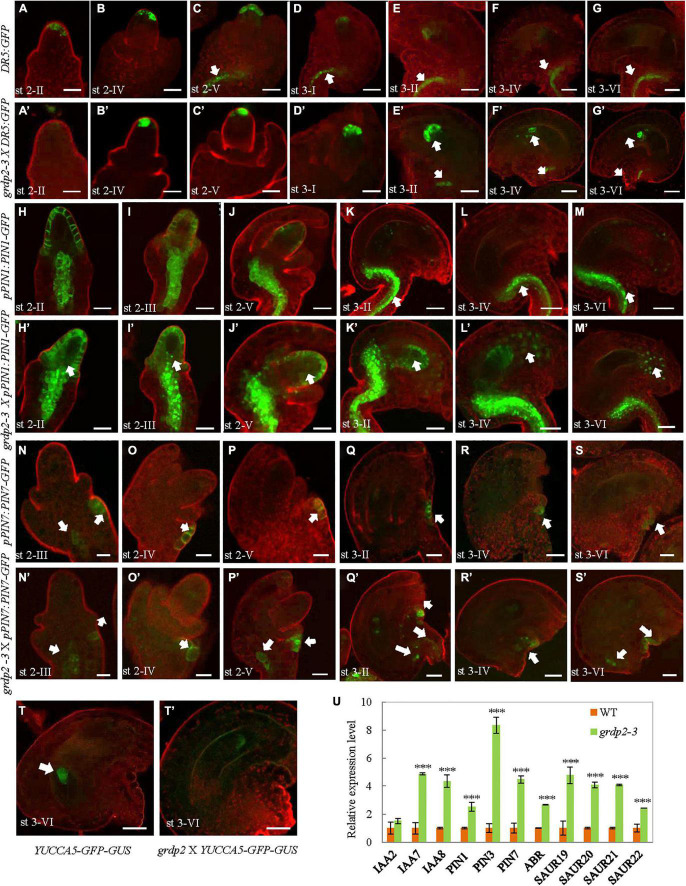
Auxin distribution in developing ovules from WT and *grdp2-3* siliques using *DR5:GFP*, *pPIN1:PIN1-GFP*, *pPIN7:PIN7-GFP*, and *YUCCA5-GFP-GUS* as an auxin reporter. **(A–G)** WT *DR5:GFP* ovules were analyzed at different developmental stages. **(A)** The cytoplasmatic GFP signal is first detected in the hypodermal L1 cell layer of the ovule primordium from stage 2-II, **(E)** until stage 3-II. **(C)** *DR5:GFP* signal is also detected in the pro-vascular cells of the funiculus from stage 2-V to **(G)** stage 3-VI. **(A’–G’)** *grdp2-3 DR5:GFP* ovules were analyzed at different developmental stages. **(B’)** The cytoplasmatic GFP signal is first detected in the hypodermal L1 cell layer of the ovule primordium from stage 2-III which is later than WT (**A**, stage 2-II), and **(G’)** last until the mature stage 3-VI. White arrows in **(E’–G’)** showed *DR5:GFP* signal in the pro-vascular cells of the funiculus is also later than WT. **(H–M)** WT *pPIN1:PIN1-GFP* ovules were analyzed at different developmental stages. **(H)** The cytoplasmatic GFP signal is first detected in the hypodermal L1 cell layer of the ovule primordium and the pro-vascular cells of the funiculus from stage 2-II, until **(J)** stage 2-V. (white arrows in **K–M**) *GFP* signal was only detected in the funiculus from stage 3-II to stage 3-IV. **(H’–M’)** *grdp2-3 pPIN1:PIN1-GFP* ovules were analyzed at different developmental stages. Except the hypodermal L1 cell layer of the ovule primordium and the pro-vascular cells of the funiculus, the GFP signal also detected in the nucellus at all developmental stages stage 2-II to stage 3-IV. **(N–S)** WT *pPIN7:PIN7-GFP* ovules were analyzed at different developmental stages. **(N)** The cytoplasmatic GFP signal is only detected in the outer integument of the ovule primordium from stage 2-III, **(S)** until stage 3-VI. **(N’–S’)** *grdp2-3 pPIN7:PIN7-GFP* ovules were analyzed at different developmental stages. **(N’)** The cytoplasmatic GFP signal can be detected not only in the outer integument of the ovule primordium but also the pro-vascular cells of the funiculus from 2-III, until **(S’)** stage 3-VI. **(T)** *YUC5:GFP-GUS* is expressed only in the chalaza at stage 3-VI. **(T’)** *grdp2-3 YUC5:GFP-GUS* ovules were analyzed at stage 3-VI, the GFP expression in the embryo sac which is a big difference with WT. In **(A–T)** and **(A’–T’)**, cell membranes were stained with FMH 4-64 FX. Scale bars = 25 μm. **(U)** Relative mRNA levels of auxin-related genes by qRT-PCR. Data are means ± SD (*n* = 3 biological replicates; ***p < 0.01, Student’s *t*-test).

Previous studies have shown that auxin plays an essential role in plant growth and female gametophyte development (Ceccato et al., 2013; Kneuper et al., 2020; Lv et al., 2020). To determine whether auxin distribution is altered in the *grdp2-3* mutant, we crossed the *DR5:GFP* reporter line and examined the spatial distribution of auxin (Benkova et al., 2003) in the *grdp2-3* plants. In the WT ovules, the weak DR5:GFP signal was detected in the epidermal L1 cell layer of the ovule primordium starting from stage 2-II (Figure 7A), and it got stronger in stage 2-IV (Figure 7B). From stage 2-V, the auxin response was detected in the epidermal cell and the funiculus pro-vascular cells until stage 3-II (Figures 7C–E). While after stage 3-IV, the DR5:GFP signal could only be found in the funiculus (Figures 7F,G). In contrast, in the *grdp2-3* ovules, the DR5:GFP signal pattern was significantly disrupted, and the signal appeared later than that of WT in the epidermal L1 cell layer of the ovule primordium starting from stage 2-IV (Figure 7B’). The DR5 signal continued until the mature stage 3-VI (Figure 7G’), and in the funiculus, DR5:GFP signal appeared later than WT (Figures 7E’–G’).

To analyze if the spatial discrepancies in ovules may be facilitated by polar auxin transport (PAT) away from the sites of synthesis, we assessed the expression of members of the PINFORMED (PIN) family of auxin efflux carriers (Friml et al., 2003). Both *pPIN1*:*PIN1*-GFP, and *pPIN7*:*PIN7*-GFP constructs were used to detect different female gametophyte development stages in the WT and *grdp2-3* backgrounds. The result showed that PIN1-GFP was strongly expressed in the chalaza and the epidermal cell at stage 2-II in WT ovules (Figure 7H). During the subsequent development stages, the PIN1-GFP signals at the epidermal cell were getting weaker and weaker, and no signal was detected at the mature stage. Simultaneously, the PIN1-GFP had a strong expression at all stages in the chalaza and funiculus (Figures 7I–M). However, the expression of PIN1-GFP was more robust in *grdp2-3* ovules and appeared in the nucellus except for the epidermal cell and chalaza (Figures 7H’,I’). Furthermore, the micropylar signal existed in the subsequent development stages in the WT (Figures 7J’–M’). The *pPIN7*:*PIN7*-GFP expression pattern did not change during the megasporogenesis (Figures 7N,O,N’,O’); however, there was a considerable difference in PIN7 expression during megagametogenesis between WT and *grdp2-3*. In addition to the outer integument (Figures 7P–S,P’–S’), *pPIN7*:*PIN7*-GFP signal was detected in the chalaza (Figures 7P’–S’). In addition, compared with the membrane-localized PIN1 and *PIN7* in WT, the signals were detected in the cytoplasm in *grdp2-3* (Figures 7H,H’,I,I’,O,P,O’,P’). Consistently, an auxin bio-synthesis gene *pYUCCA5-GFP-GUS* was used to detect the auxin synthesis in *grdp2-3* ovules. The result showed that auxin biosynthesis at stage 3-VI ovules of *grdp2-3* was misregulated (Figures 7T,T’). Also, we found both the patterns and intensity of *DR5, PIN1*, *PIN2*, and *PIN7* were altered in *grdp2-3* roots (Supplementary Figure 1). These changes in expression patterns of auxin response and bio-synthesis genes suggest that auxin biosynthesis and transport may be affected in *grdp2-3.*

We further determined the altered auxin signaling and transport-related defects in *grdp2* by analyzing the transcript levels of auxin-related genes in WT and *grdp2-3* by qRT-PCR. The auxin efflux carriers *PIN1*, *PIN3*, *PIN7*, and *ABR*; auxin-responsive genes *SAUR19*, *SAUR20*, *SAUR21*, and *SAUR22*, and auxin signaling genes *IAA2*, *IAA7*, and *IAA8* were analyzed. For most of the genes, there was a significant increase in their transcript level in the inflorescence of *grdp2*-3, and no substantial increase was detected for *IAA2* (Figure 7U). We further studied the auxin response in the *grdp2-3* mutants by exogenously applying the auxin (IAA) and auxin transport inhibitor (NPA) and observed the growth of the *grdp2-3* roots after treatment. The results indicate that auxin accumulation in *grdp2-3* results in more rapid root growth (Supplementary Figure 2). These observations suggest that the auxin signaling and transport are disrupted in the *grdp2* mutant.

### Overexpression of *GRDP1* Results in a Similar Phenotype Like *GRDP2*

**FIGURE 8:**
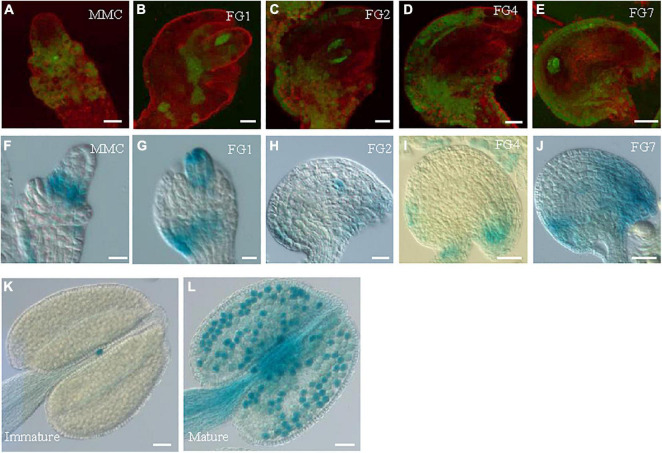
The expression profiles of *GRDP1* in *Arabidopsis.* **(A–E)** *pGRDP1:GFP* expression pattern in different ovule development stages. The developmental stages of the ovules are indicated at the top right corner of each panel. Bars = 20 μm. **(F–J)** *pGRDP1:GUS* expression pattern in ovules. Bars = 20 μm. **(K,L)** *pGRDP1:GUS* expression pattern in pollen. Bars = 50 μm.

*GRDP1* and *GRDP2* are the two members of GRDPs in *Arabidopsis*. Their protein sequences share 64% identity (Rodriguez-Hernandez et al., 2017). Therefore, we speculated that there could be similarities in gene function between the two genes. The *grdp1* T-DNA (SALK_079708) insertion site and *GRDP1* expression level are shown in Supplementary Figure 3A. The development of the seed in *grdp1* mutant was normal (Supplementary Figures 3B,C). We fused the *GRDP1* promoter with GFP and GUS protein to detect the expression patterns of *GRDP1* in different stages of ovule development. The results showed that GFP and GUS signals have the same expression pattern during ovule development. At first, the signals appear in nucellus tissues at the MMC stage (Figures 8A,F). With the development, the signals can be detected in both the embryo sac and nucellus tissue from FG1 to FG2 stage (Figures 8B,C,G,H). During the subsequent developmental stages, the signals appeared at the chalaza end and in integument tissues (Figures 8D,E,I,J). We also detected the expression pattern of *GRDP1* in pollen and found that the *GRDP1* expressed in mature anther (Figures 8K,L), which is different from the expression pattern of *GRDP2*. We also use the CRISPR/Cas9 genome-editing system to get the *grdp1grdp2* double mutant (Wang and Chen, 2019), the result showed that the seed setting is normal in *Crispr-7^#^* (*grdp1grdp2* double mutant) line (Supplementary Figures 4A–D).

**FIGURE 9:**
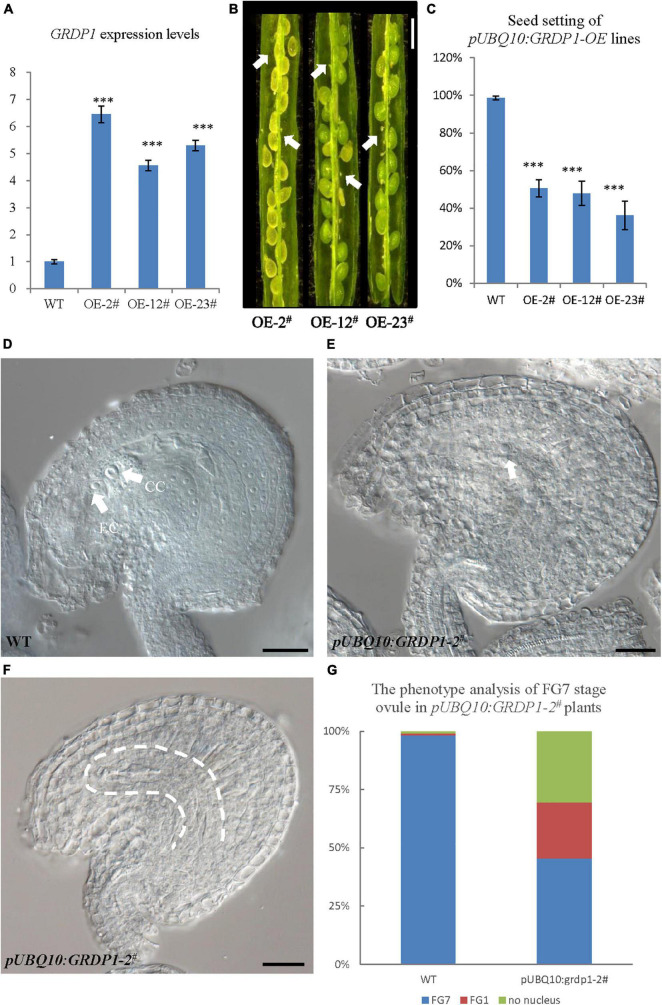
Phenotype analysis of *pUBQ10:GRDP1*-OE lines. **(A)** Expression levels of *GRDP1* in WT, *pUBQ10:GRDP1-OE-2^#^*, *pUBQ10:GRDP1-OE-12^#^*, and *pUBQ10:GRDP1-OE-23^#^* as determined by real-time RT-PCR. **(B)** Seed development in three *pUBQ10:GRDP1-OE* line plants. Bar = 0.5 cm. **(C)** Seed setting statistics of the WT, *pUBQ10:GRDP1-OE-2^#^*, *pUBQ10:GRDP1-OE-12^#^*, and *pUBQ10:GRDP1-OE-23^#^* lines. **(D)** The FG7 stage ovules from WT plants. **(E,F)** The FG7 stage ovules from *pUBQ10:GRDP1-OE-2^#^* plants, 24.1% of 196 examined FG7 stage ovules showed single nucleus, 30.6% of 196 examined FG7 stage ovules showed no nucleus. **(G)** The phenotype analysis of FG7 stage ovules in WT and *pUBQ10:GRDP1-OE-2^#^* plants. Vertical bars represent the mean ± SD as determined by two technical replicates of three biological replicates; asterisks show the significance level judged by Student’s *t*-test (^∗∗∗^*p* < 0.01). CC, central cell; EC, egg cell. White arrows mean nuclei; white dotted lines mean embryo sac.

The overexpression lines using *UBQ10* derived *GRDP1* (*pUBQ10:GRDP1-OE-2^#^, pUBQ10:GRDP1-OE-12^#^*, and *pUBQ10:GRDP1-OE-23^#^*) showed significant up-regulation in *GRDP1* expression (Figure 9A). The seed setting was decreased considerably (50.5%,47.8%, and 36.2%, respectively) in *GRDP1* overexpressing lines (Figures 9B,C). Further phenotypic observation of the ovules of *pUBQ10:GRDP1-OE-2^#^* plants at FG7 stage showed that there were two types of abortions; 30.6% of the ovules had no embryo sac, and 24.1% of the ovules were arrested at FG1 stage (Figures 9D–G). These results indicate that *GRDP1* and *GRDP2* have functional redundancy in the regulation of ovule development.

## Discussion

The canonical glycine-rich proteins contain a high glycine percentage (from 40% to 70%), with arranged (Gly)n-X repetitions (Sachetto-Martins et al., 2000; Mousavi and Hotta, 2005). Besides the canonical GRPs, there are GRDPs containing a short glycine-rich region. Several transcripts encoding GRDPs have been reported to be induced under abiotic stress (Bocca et al., 2005; Ortega-Amaro et al., 2014; Rodríguez-Hernández et al., 2014). The expression patterns of some canonical plant GRPs have been reported in floral organs. The oleosin-like protein GRP17 was identified as a component of the pollen wall of *Arabidopsis thaliana* and is needed for rapid initiation of pollination (Suzuki et al., 1999; Mayfield and Preuss, 2000). Another GRP, GRP23, is a novel nuclear PPR domain protein critical for early embryogenesis (Ding et al., 2006). Transcripts of *Arabidopsis* GRPs (*GRP1* and *GRP2*) express abundantly in flowers, suggesting their essential functions in floral organ development (de Oliveira et al., 1990). These studies indicate that GRPs play a variety of roles in developing flower organs, male and female gametophytes. In this study, we describe the *GRDP2* gene function in female gametophyte development. *GRDP2* encodes a non-canonical glycine-rich protein of unknown function containing a DUF1399 domain, a putative RNA-binding motif, and a glycine-rich domain. The *GRDP2* gene is regulated, with exceptionally high mRNA levels in inflorescence than root, stem, leaf, and immature siliques, implying that *GRDP2* participate in plant reproductive development process (Figure 2A). The different expression patterns of GUS or GFP in ovules and pollen indicate the different functions of *GRDP2* in male or female gametophytes development.

The phenotype analysis of the three mutant alleles *grdp2-1*, *grdp2-2*, and *grdp2-3* shared different developmental defects in seed sets, indicating the significant difference between the expression level of *GRDP2* in different alleles may account for severe phenotypes in seed sets of *grdp2-3*. Our results showed that the expression level of *GRDP2* in *grdp2-1* and *grdp2-2* mutants were knockout or knockdown, but the expression level of *GRDP2* in *grdp2-3* was significantly increased (Figures 1A,B). We further found that *grdp2-3* overexpression lines caused reduced plant fertility (50%), but the pollen development was not affected (Figures 1C–E). The female gametogenesis of *grdp2-3* mutant was disrupted with abnormal embryo sac degradation and nuclear number at the FG7 stage (Figures 2K,L). Similar to *grdp2-3*, the *p35S:GRDP2-OE* and *pUBQ10:GRDP2-OE* overexpression lines exhibited low seed sets and female gametophyte abortion (Figure 4). These data show that *GRDP2* overexpression leads to abnormal female gametogenesis. DIC observation (Figures 3H,I), and callose staining (Figures 5C–E) showed that the megasporogenesis process was also affected by the overexpression of *GRDP2*. Unlike the typical GRP proteins, the *GRDP2* function is mainly in the female gametophyte development process. This may be due to the differences in the structure of the glycine-rich domain.

Hormones, such as auxins, are involved in the complex molecular network that regulates the coordinated development of plant organs (Bennett et al., 1996; Benkova et al., 2003). Genes controlling ovule patterning have been identified and studied in detail (Qin et al., 2014; Zhao et al., 2018; Su et al., 2020); however, the roles of auxin in ovule development are largely unknown. Based on our data on local auxin synthesis, PIN localization, and auxin response patterns, we found that overexpression of *GRDP2* leads to defective auxin distribution in the whole ovule development process (Figure 7). Interestingly, it has been reported that the *GRDP2* belongs to a group of genes regulated explicitly by indole-3-acetic acid (IAA) (Goda et al., 2004). We analyzed IAA expression levels in WT and *grdp2-3* lines, our result of qRT-PCR verified that *grdp2-3* lines accumulated the higher auxin levels (Figure 7U). The abnormal auxin responses at the embryo sac might explain defective embryo sac development in *grdp2-3* because auxin is critical for embryo sac patterning and gamete specification (Pagnussat et al., 2009). Our results reveal that overexpression of *GRDP2* results in abnormal ovule development possibly through an auxin-dependent mechanism.

The different expression patterns of *GRDP1* (Figures 8K,L) and *GRDP2* (Figures 2C,D) in anther indicate that they may have different roles in pollen development. However, the overexpression lines of *pUBQ10:GRDP1* show two types of abnormal ovules at FG1 stage and FG7 stage (Figures 9D–G), which is similar to *GRDP2* overexpression plants suggesting that *GRDP1* and *GRDP2* could have redundant functions during ovule development in *Arabidopsis thaliana*.

Taken together, our results indicate that the excess accumulation of GRDP2 is harmful to ovule development in *Arabidopsis*, resulting in disorder of auxin distribution in the ovule and abnormal ovule development.

## Data Availability Statement

The original contributions presented in the study are included in the article/Supplementary Material, further inquiries can be directed to the corresponding authors.

## Author Contributions

LW performed most of the experiments, analyzed the research results, and wrote the manuscript. YL and BJ contributed to data analysis. HC, MA, and YQ made the critical revision. All authors contributed to the article and approved the submitted version.

## Conflict of Interest

The authors declare that the research was conducted in the absence of any commercial or financial relationships that could be construed as a potential conflict of interest.

## Publisher’s Note

All claims expressed in this article are solely those of the authors and do not necessarily represent those of their affiliated organizations, or those of the publisher, the editors and the reviewers. Any product that may be evaluated in this article, or claim that may be made by its manufacturer, is not guaranteed or endorsed by the publisher.

## Footnotes

1
https://abrc.osu.edu/

2
http://signal.salk.edu/

3
http://crispr.dfci.harvard.edu/SSC/
