# Supplementary figures and images for: The Glycine-Rich Domain Protein GRDP2 Regulates Ovule Development via the Auxin Pathway in Arabidopsis
Source: Front Plant Sci. 2021 Oct 29;12:698487. doi: 10.3389/fpls.2021.698487 (PMC8585784; doi:10.3389/fpls.2021.698487)

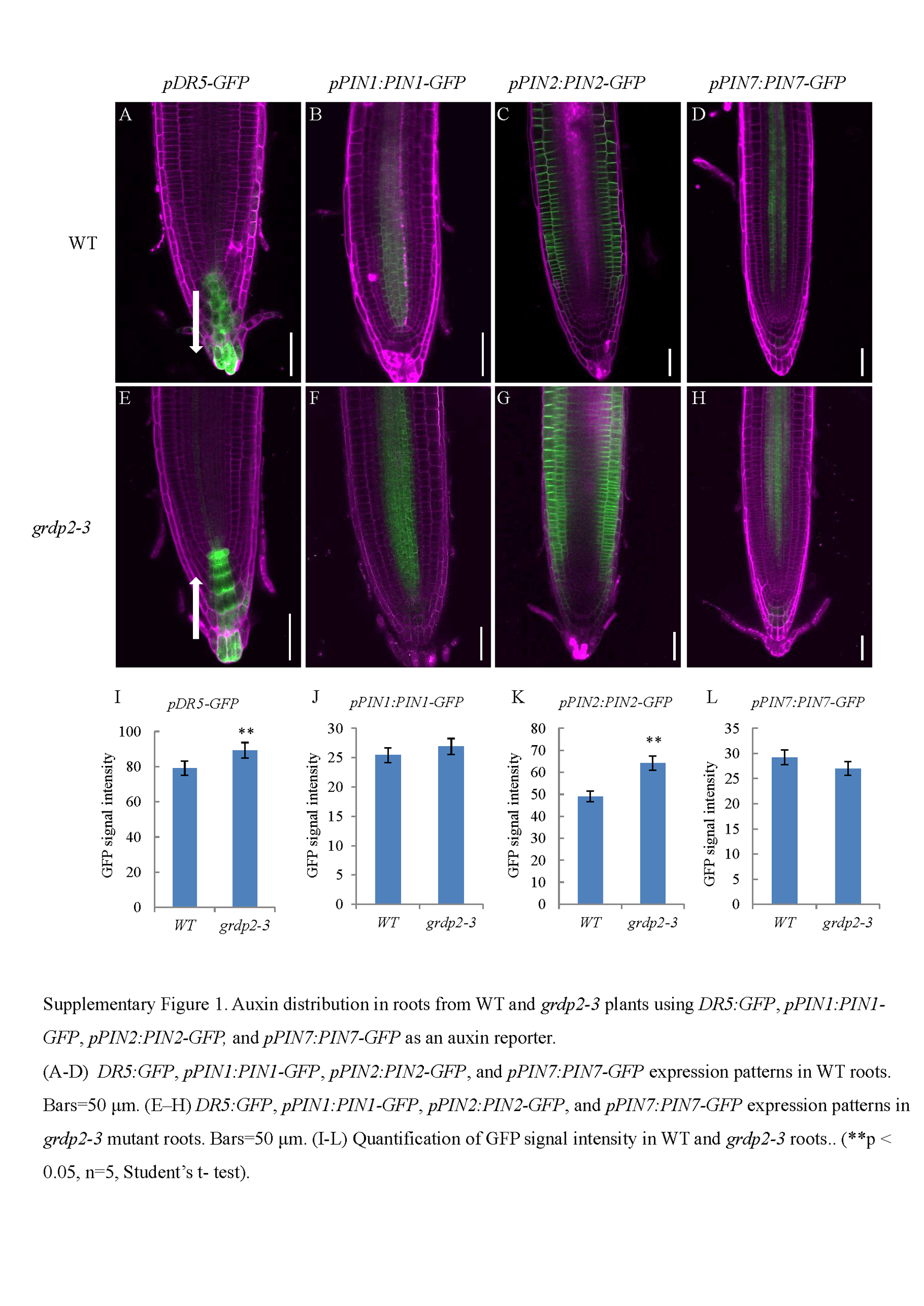

Supplement: Supplementary file 1 [file Image_1.TIFF]

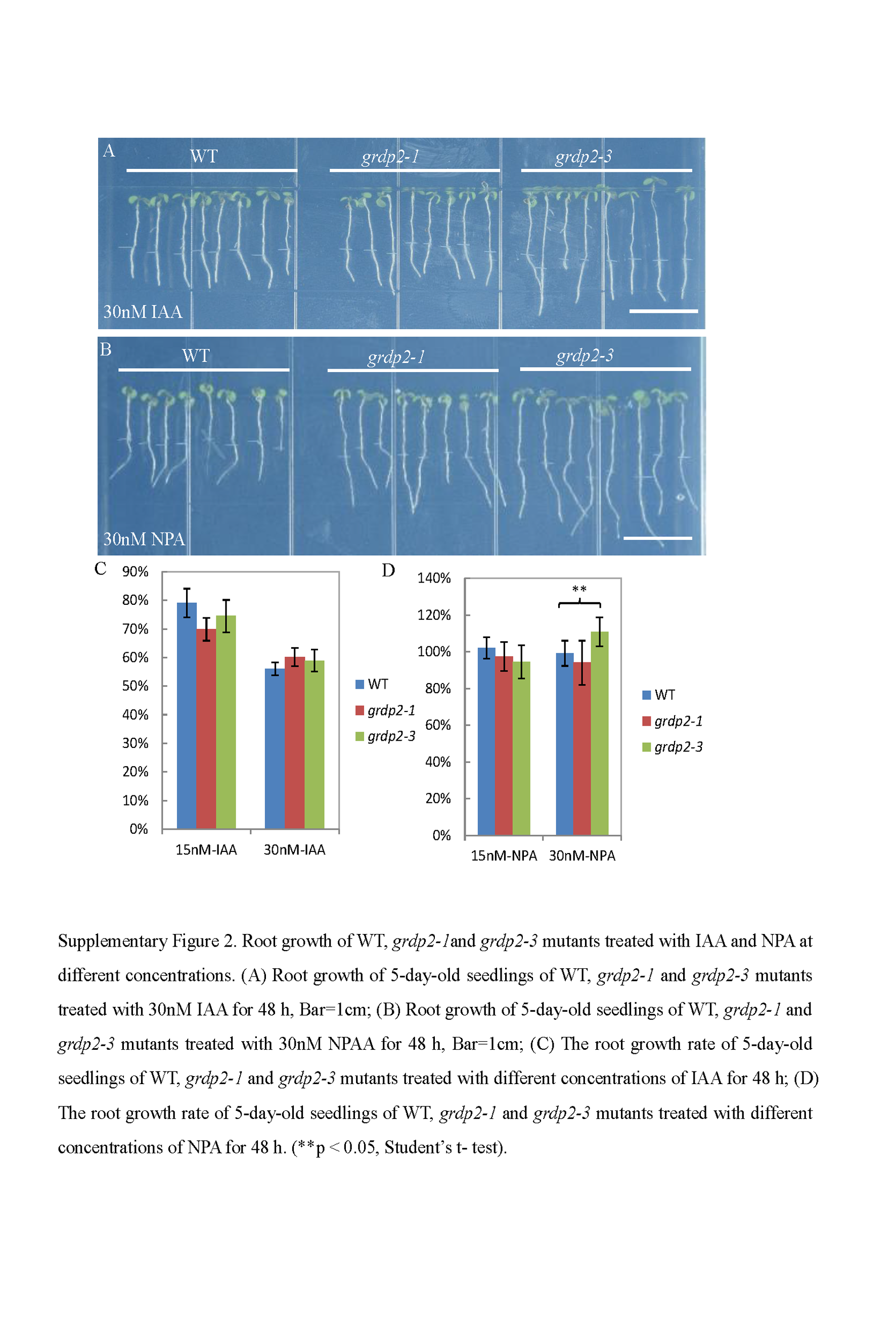

Supplement: Supplementary file 2 [file Image_2.TIFF]

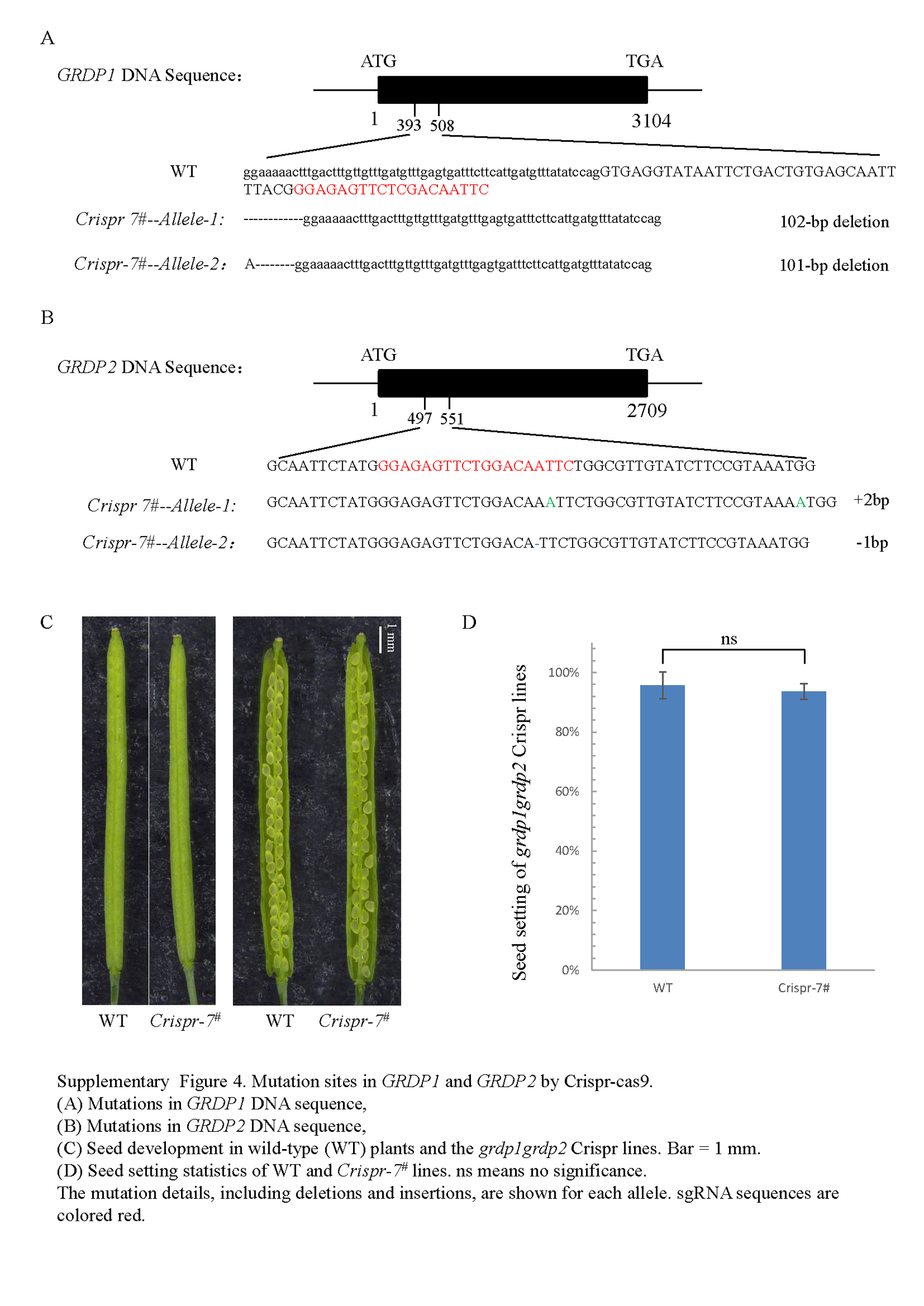

Supplement: Supplementary file 3 [file Image_3.TIFF]

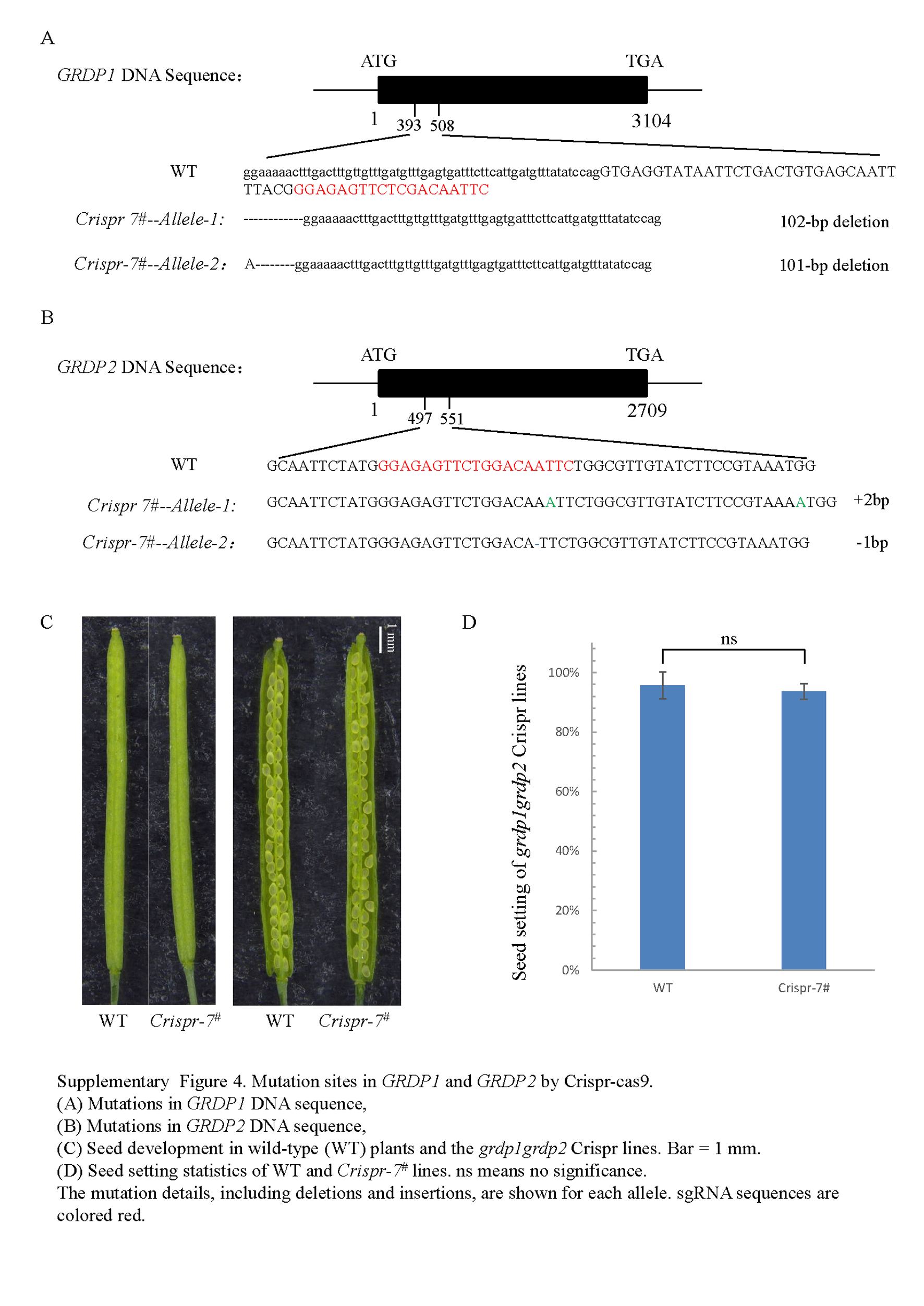

Supplement: Supplementary file 4 [file Image_4.TIFF]
